# Apoptosis signal-regulating kinase 1 mediates the inhibitory effect of hepatocyte nuclear factor-4α on hepatocellular carcinoma

**DOI:** 10.18632/oncotarget.8478

**Published:** 2016-03-30

**Authors:** Cai-Feng Jiang, Liang-Zhi Wen, Chuan Yin, Wen-Ping Xu, Bin Shi, Xin Zhang, Wei-Fen Xie

**Affiliations:** ^1^ Department of Gastroenterology, Changzheng Hospital, Second Military Medical University, Shanghai 200003, China

**Keywords:** apoptosis signal-regulating kinase 1, hepatocyte nuclear factor 4α, hepatocellular carcinoma, mitogen-activated protein kinases, differentiation therapy

## Abstract

Previous studies provided substantial evidence of a striking suppressive effect of hepatocyte nuclear factor 4α (HNF4α) on hepatocellular carcinoma (HCC). Apoptosis signal-regulating kinase 1 (ASK1) is involved in death receptor-mediated apoptosis and may acts as a tumor suppressor in hepatocarcinogenesis. However, the status and function of ASK1 during HCC progression are unclear. In this study, we found that HNF4α increased ASK1 expression by directly binding to its promoter. ASK1 expression was dramatically suppressed and correlated with HNF4α levels in HCC tissues. Reduced ASK1 expression was associated with aggressive tumors and poor prognosis for human HCC. Moreover, ASK1 inhibited the malignant phenotype of HCC cells *in vitro*. Intratumoral ASK1 injection significantly suppressed the growth of subcutaneous HCC xenografts in nude mice. More interestingly, systemic ASK1 delivery strikingly inhibited the growth of orthotopic HCC nodules in NOD/SCID mice. In addition, inhibition of endogenous ASK1 partially reversed the suppressive effects of HNF4α on HCC. Collectively, this study highlights the suppressive effect of ASK1 on HCC and its biological significance in HCC development. These outcomes broaden the knowledge of ASK1 function in HCC progression, and provide a novel potential prognostic biomarker and therapeutic target for advanced HCC.

## INTRODUCTION

Hepatocellular carcinoma (HCC) is one of the most common malignant tumors worldwide [1]. Although progression of medical technology has led to great developments in HCC diagnosis and treatment, the long-term survival of HCC patients remains unsatisfactory [2]. Hence, strategies to inhibit HCC progression are urgently needed.

Hepatocyte nuclear factor 4α (HNF4α), a principal member of the hepatic transcription factor network, plays an indispensable role in regulating hepatic lineage differentiation and in maintaining liver function [3, 4]. It is also linked to a variety of human diseases including diabetes mellitus, colitis, and cancer [5–8]. It has been reported that transient suppression of HNF4α initiates hepatocellular transformation [9] and HNF4α knockout in adult hepatocytes leads to hepatocyte proliferation and promotes the development of HCC in mice [10], suggesting that HNF4α plays a critical role in liver carcinogenesis. Our previous studies indicate that HNF4α expression is decreased gradually during hepatocarcinogenesis after diethylnitrosamine (DEN) administration in rats [11] and that reduced HNF4α expression is associated with aggressive behavior and poor prognosis in HCC patients [12]. Furthermore, forced HNF4α overexpression induced the hepatoma cell differentiation into hepatocytes and suppressed *in vivo* HCC growth and metastasis [12, 13]. In addition, HNF4α administration attenuated liver fibrosis in rats [14]. More recently, molecular mechanisms, such as miRNA cascades and inflammatory signaling pathways, through which HNF4α inhibits HCC have been clarified [9, 12, 15, 16]. However, as a key hepatic transcription factor, whether other signaling pathways are involved in the mechanisms of HNF4α need for further study.

Apoptosis signal-regulating kinase 1 (ASK1), also known as mitogen-activated protein 3K5, is a member of the mitogen-activated protein kinase kinase kinase (MAP3K) family that selectively activates downstream mitogen-activated protein kinases (MAPKs), c-Jun N-terminal kinases (JNKs), and p38 MAPKs in response to various cellular stresses [17, 18]. In particular, ASK1 has been identified as a key determiner of cell death via triggering cell apoptosis. Interestingly, ASK1 has also been reported to promote cellular differentiation. Recent studies revealed that ASK1 may be involved in differentiation process in diverse cell types, including keratinocytes [19], chondrocytes [20] and stem cells [21]. On the other hand, substantial evidence demonstrate that a number of cancers are intimately related to ASK1 mediated cascades [22–24]. However, the role of ASK1 in malignances remains controversial [25–30]. Nakagawa *et al*. showed that ASK1-deficient mice had increased susceptibility to DEN-induced HCC [31]. It was also reported that ASK1-p38 MAPK signaling was implicated in DEN-induced hepatocellular carcinogenesis in Toll-like receptor 2-deficient mice [32]. These data suggest that ASK1 plays an important role in hepatocarcinogenesis, yet the role for ASK1 as a drug target in HCC and the significance of ASK1 expression in patients are obscure.

In this study, we demonstrate the prognostic value of ASK1 in HCC patients and the inhibitory effect on HCC progression both *in vitro* and *in vivo*. These results not only deepen our understanding of the biological significance of ASK1 but also provide a promising new therapeutic target and a novel prognostic biomarker for HCC.

## RESULTS

### HNF4α regulates MAPK signaling and activates ASK1 expression

**Figure 1:**
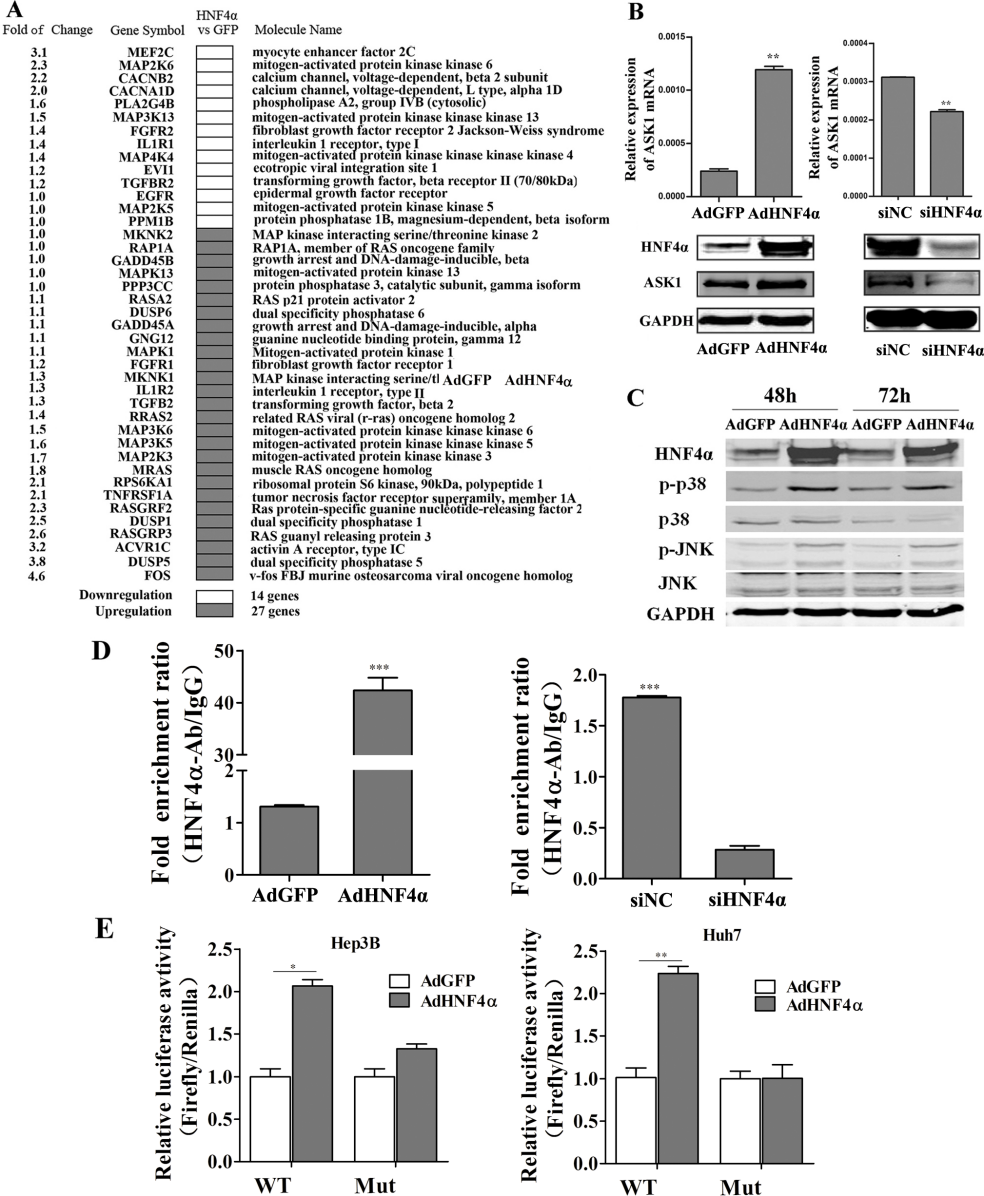
HNF4α regulates the MAPK signaling pathway and activates ASK1 by binding to its promoter (**A**) cDNA microarray analysis identified downregulated and upregulated MAPK signaling pathway genes in Hep3B cells infected with AdHNF4α or AdGFP after 72 h. (**B**) RT-PCR and Western blot analysis of ASK1 expression in AdHNF4α-infected or siHNF4α-transfected Hep3B cells after 72 h. (**C**) Western blot analysis of phospho- (p-)p38, p38, p-JNK, and JNK expression in AdHNF4α-infected Hep3B cells after 48 h and 72 h. (**D**) ChIP assays revealed HNF4α binding to *ASK1* promoter region. RT-PCR was performed to examine DNA fragments immunoprecipitated by the anti-HNF4α antibody with primers targeting the HNF4α response element. DNA immunoprecipitated using normal rabbit IgG (IgG) was used as the control. (**E**) Luciferase activity in HCC cells for a reporter plasmid harboring the HNF4α response element derived from the *ASK1* promoter. Mutation of the HNF4α response element (Mut) abolished HNF4α transcriptional activity. Each value represents the mean ± SD of triplicate experiments. Data represent the mean ± SD, *P < 0.05, **P < 0.01.

HNF4α is regarded as a key suppressor of hepatic carcinoma. To explore the mechanisms underlying the inhibitory effect of HNF4α on HCC, a cDNA microarray was performed to obtain gene expression profiles in Hep3B cells after recombinant adenoviruses AdHNF4α infection to induce HNF4α overexpression. HNF4α positively regulated 1218 mRNAs and negatively regulated 1411 mRNAs for more than 2 times (The entire dataset is available at NCBI Gene Expression Omnibus http://www.ncbi.nlm.nih.gov/geo/ with accession number GSE66785). Pathway analysis using the DAVID database [33] showed that many differentially expressed genes were known to be involved in hepatocyte function, such as complement and coagulation cascades, metabolism, Type 2 diabetes mellitus, etc. Interestingly, a cluster of genes in cancer-associated pathways, such as the p53 and MAPK signaling pathways, were also regulated by HNF4α (Supplementary Figure S1). As the MAPK signal transduction pathway is crucial for determining cell differentiation [19], we then focused on its role in HNF4α-mediated cell differentiation. In total, 41 genes in the MAPK signaling pathway were differentially regulated by HNF4α in Hep3B cells. 14 genes were downregulated and 27 were upregulated (Figure 1A). The significant increase in *ASK1* expression was particular interesting because it is a key mediator of MAPK signaling and is reported to be involved in the pathogenesis of many tumors. We then validated the effect of HNF4α on *ASK1* expression by real-time polymerase chain reaction (RT-PCR) and Western blotting. ASK1 expression appeared to be sensitive to the level of HNF4α. It was increased by HNF4α overexpression and decreased by HNF4α knockdown (Figure 1B and Supplementary Figure S2). Consistently, Western blot analysis showed that phosphorylation of the JNK and p38 (MAPKs downstream of ASK1) were also increased after HNF4α overexpression (Figure 1C). This result confirmed that MAPKs can be activated by HNF4α. We then used the JASPAR database [34] to predict the site of HNF4α response element (HNF4α-RE) in the promoter region of *ASK1* gene. One HNF4α-RE was identified when the profile score threshold was set to 80% (Supplementary Figure S3); this was confirmed by chromatin immunoprecipitation (ChIP) assay. As shown in Figure 1D, the binding of HNF4α to *ASK1* promoter was highly enriched in Hep3B cells with HNF4α overexpression. In contrast, knockdown of HNF4α by small interfering RNA (siRNA) in Hep3B cells substantially decreased the binding enrichment. These data suggest direct binding between endogenous ASK1 and HNF4α in HCC cells. To further determine the effect of HNF4α on *ASK1* transactivation, luciferase reporter plasmids containing the *ASK1* promoter with the HNF4α-RE were transfected into AdHNF4α-infected Hep3B and Huh7 cells. The reporter assay showed that ectopic HNF4α expression increased the transcriptional activity of *ASK1* promoter, and that this effect was significantly impaired by mutation of the HNF4α-RE (Figure 1E and Supplementary Table S1). Together, these data reveal that HNF4α activates *ASK1* transcription by binding to its promoter.

### Reduced ASK1 expression is associated with aggressive clinicopathological features and poor prognosis for human HCC

**Figure 2:**
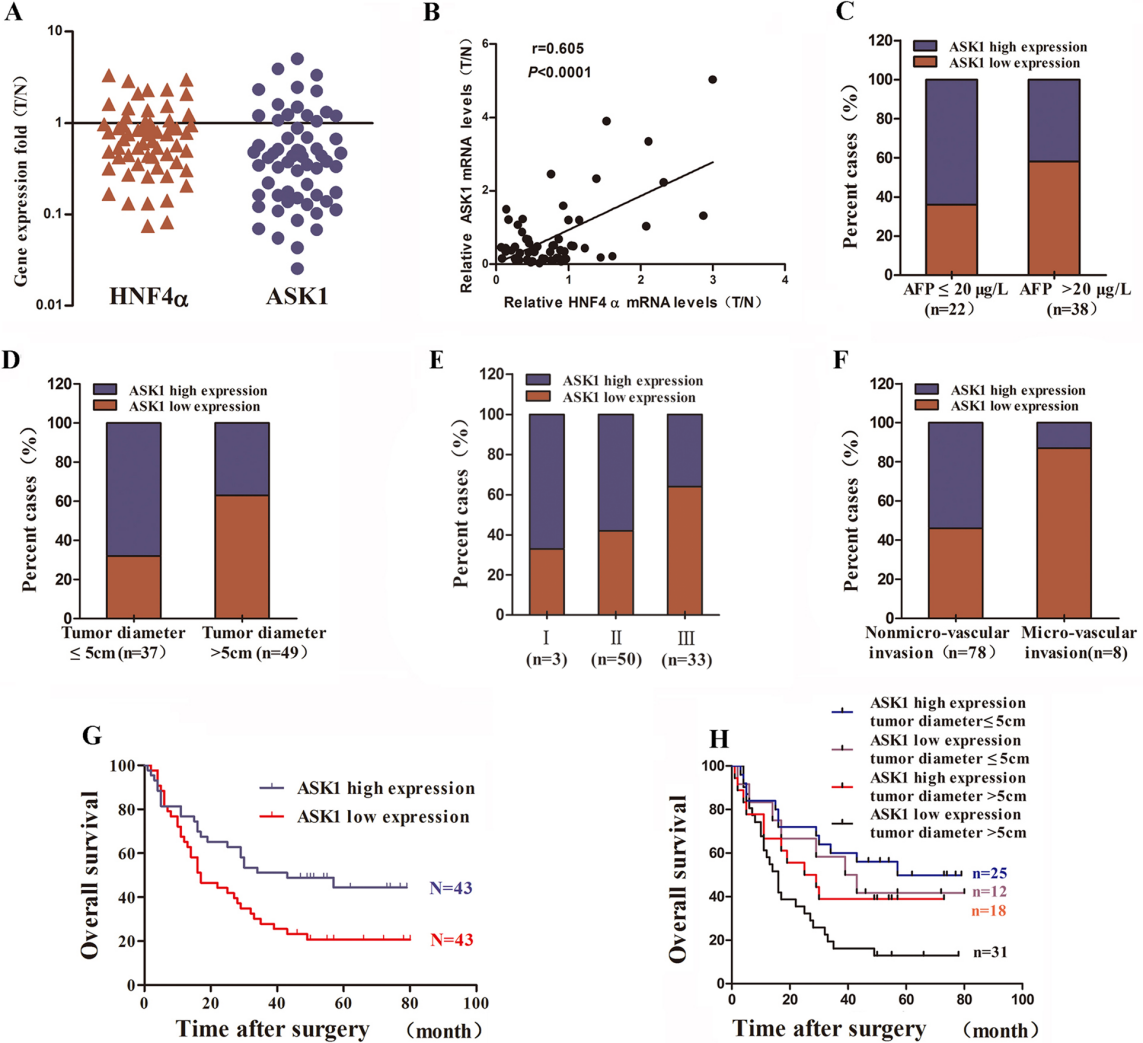
Reduced ASK1 expression is associated with aggressive clinicopathological features and poor prognosis of human HCC (**A**) HNF4α and ASK1 mRNA levels in human HCC samples (*n* = 60), as detected by RT-PCR. The results are presented as the fold change in gene expression in HCC tissue (T) versus noncancerous adjacent tissue (N). (**B**) The positive correlation between HNF4α and ASK1 expression in HCC subjects (*r* = 0.605, *P* < 0.0001, *n* = 60). (**C**) Reduced ASK1 mRNA expression was more common in HCC samples with high AFP mRNA levels than in those with low AFP mRNA levels. Low level: AFP ≤ 20 μg/L, *n* = 22; high level: AFP > 20 μg/L, *n* = 38. (**D**–**F**) Reduced ASK1 protein level is more common in samples from HCC with more aggressive pathological features, including a larger tumor size (D), advanced tumor stage (E), and more microvascular invasion (F). (**G**) Kaplan–Meier analysis of the overall survival of 86 HCC patients. The median ASK1 protein level for all 86 HCC samples was chosen as the cutoff point. *P* = 0.0116 by the log-rank test. (**H**) A combination of ASK1 protein level and tumor diameter enhanced the prognostic accuracy in HCC patients.

We next examined ASK1 and HNF4α mRNA levels in HCC tissue specimens and their surrounding noncancerous tissue (NT) from 60 patients (defined as Group 1) by RT-PCR. Compared with NT, HNF4α mRNA was downregulated in 45 of 60 cases (75%) and ASK1 mRNA was downregulated in 44 of 60 cases (73.33%; Figure 2A). Moreover, ASK1 expression was positively correlated with HNF4α levels in HCC patients (*r* = 0.605, *P* < 0.0001; Figure 2B). The clinicopathological significance of ASK1 and HNF4α expression was further analyzed. The median mRNA level of ASK1 and HNF4α was chosen as the cutoff point, leaving 30 cases in each group (Supplementary Tables S2–S3). ASK1 and HNF4α mRNA levels were inversely correlated with α-fetoprotein (AFP) mRNA levels (Figure 2C, *P* = 0.0028 and Supplementary Figure S4A). More interestingly, low ASK1 mRNA expression was also associated with an aggressive HCC phenotype, including larger tumor size (*P* = 0.034), advanced tumor stage (*P* = 0.009) and an absence of tumor encapsulation (*P* = 0.013; Supplementary Table S2). Consistently, reduced HNF4α levels correlated well with larger tumor size (*P* = 0.0001) and advanced tumor stage (*P* = 0.022; Supplementary Table S3). In addition, low ASK1 and HNF4α expression were more likely in human HCC specimens containing high levels of AFP, tumor diameter > 5 cm and advanced tumor stage, vice versa (Supplementary Figure S4B–S4D).

We then examined the levels of HNF4α, ASK1 and p-ASK1 in three HCC clinical samples by immunohistochemistry. As shown in Supplementary Figure S5, HNF4α was significantly decreased in HCC. Consistently, both ASK1 and p-ASK1 were also reduced in HCC compared with NT, further suggested that the expression of AKS1 was correlated with HNF4α. As ASK1 is activated by phosphorylation, the data also indicated that the activity of ASK1 also reduced in HCC.

To further determine whether ASK1 low-expression predicts poor prognosis of HCC patients, a HCC tissue microarray was performed to detect ASK1 protein level. Consistent with the data of ASK1 mRNA levels in Group 1, ASK1 protein levels was downregulated in 53 of 86 cases (61.63%) compared with NT. The median expression value of ASK1 protein was used as the cut-off to separate patients into groups with high or low ASK1 levels. Furthermore, reduced ASK1 protein expression was more frequent in HCCs with a tumor diameter > 5 cm than in those with a tumor diameter ≤ 5 cm (63% versus 32%; Figure 2D, *P* < 0.0001). In addition, patients with an advanced tumor stage or microvascular invasion showed relatively low ASK1 protein levels (Figure 2E, *P* < 0.0001; and Figure 2F, *P* < 0.0001) and patients with low ASK1 expression exhibited a much lower overall survival (OS) compared with those with high ASK1 expression (median OS 22 and 47 months, respectively; *P* = 0.0116; Figure 2G). More interestingly, ASK1 expression combined with tumor diameter dramatically enhanced the predictive accuracy of prognosis (Figure 2H, *P* = 0.0095).

### ASK1 inhibits HCC cell malignancy *in vitro*

**Figure 3:**
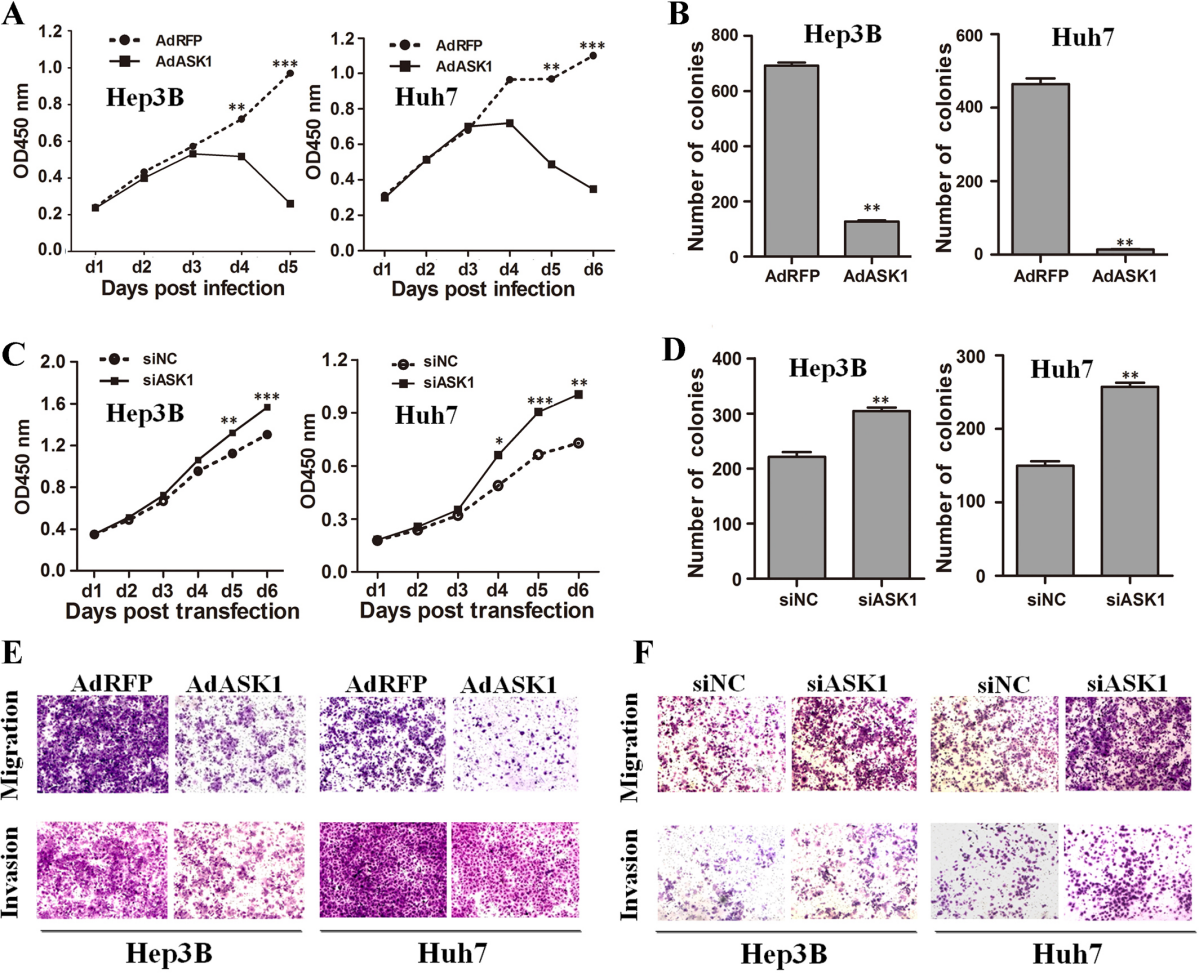
ASK1 represses the malignant properties of HCC cells *in vitro* (**A**) The proliferation of HCC cells infected with AdASK1 or AdRFP was measured using the Cell Counting Kit-8. (**B**) Colony formation assays were performed for Hep3B and Huh7 cells infected with AdASK1 or AdRFP. (**C**) Proliferation of Hep3B and Huh7 cells transfected with siASK1 or scramble siRNA (siNC). (**D**) Colony formation assays for Hep3B and Huh7 cells transfected with siASK1 or siNC. (**E**, **F**) Migration and invasion were assessed by transwell assays for HCC cells infected with AdASK1 or AdRFP (E) or transfected with siASK1 or siNC (F). Data represent the mean ± SD, *P < 0.05, **P < 0.01, ***P < 0.001.

We next functionally characterized ASK1 by focusing on its effect on HCC cell malignancy. HCC cells infected with recombinant adenoviruses AdASK1 showed a lower proliferation rate and fewer colonies compared with cells infected with control virus AdRFP (Figure 3A, 3B and Supplementary Figure S6A). Moreover, ASK1 overexpression induced more cell death in HCC cells, as assessed by annexin V staining (Supplementary Figure S7). In contrast, inhibition of endogenous ASK1 by siASK1 (Supplementary Figure S8) promoted cell growth and colony formation (Figure 3C, 3D and Supplementary Figure S6A). In addition, ASK1 upregulation significantly inhibited HCC cell migration and invasion (Figure 3E and Supplementary Figure S6B). In contrast, ASK1 downregulation enhanced HCC cell migration and invasion (Figure 3F and Supplementary Figure S6B).

### ASK1 abolishes tumorigenicity and suppresses growth of HCC *in vivo*

**Figure 4:**
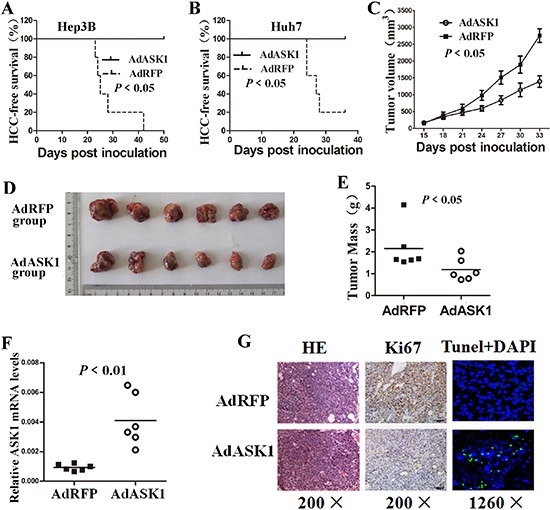
ASK1 overexpression suppresses tumorigenicity of HCC cells in mice (**A**, **B**) HCC-free survival in mice injected with AdASK1- or AdRFP-infected Hep3B (A) and Huh7 cells (B) was analyzed by the Kaplan–Meier method and compared using the log-rank test (*n* = 5). (**C**–**E**) Intratumoral AdASK1 injection repressed HCC xenograft growth. A subcutaneous liver tumor model was established based on Huh7 cell implantation. HCC xenografts were then injected with AdASK1 or AdRFP virus. Tumor size was estimated by serial calculation (C) and tumor weight was measured at the endpoint (D, E) (*n* = 6). (**F**) RT-PCR analysis of ASK1 mRNA expression in Huh7 tumor nodules injected with AdASK1 or AdRFP. (**G**) Representative images of H & E stained, Ki67 immunohistochemistry and tunel stained of serial sections from Huh7 tumor nodules injected with AdASK1 or AdRFP. Magnification, ×200 and ×1260; Data in C are shown as the mean ± SD. Horizontal lines in E and F indicate the median values.

To examine the effect of ASK1 on *in vivo* tumorigenesis, Hep3B or Huh7 cells infected with AdRFP or AdASK1 were subcutaneously transplanted into the flanks of BALB/c nude mice. Six weeks later, xenografts were detected in 80% (four out of five) mice in the AdRFP-Huh7 cell group and in 100% of Hep3B-inoculated mice. However, no xenografts were observed in mice inoculated with ASK1-infected HCC cells (Figure 4A, 4B). These results demonstrate that ASK1 overexpression can eliminate the tumorigenicity of HCC cells *in vivo*.

We then investigated the antitumor effect of ASK1 in an established HCC cell transplant model by subcutaneous injection of Huh7 cells into BALB/c nude mice. Intratumoral injection of AdASK1 significantly reduced the growth and weight of xenografts compared with the control AdRFP group (Figure 4C–4E). ASK1 mRNA expression was striking increased in AdASK1-treated tumor nodules (Figure 4F). Immunostaining and TUNEL assay showed that AdASK1 treatment resulted in striking Ki67 downregulation accompanied by more TUNEL-positive cells (Figure 4G and Supplementary Figure S9A).

**Figure 5:**
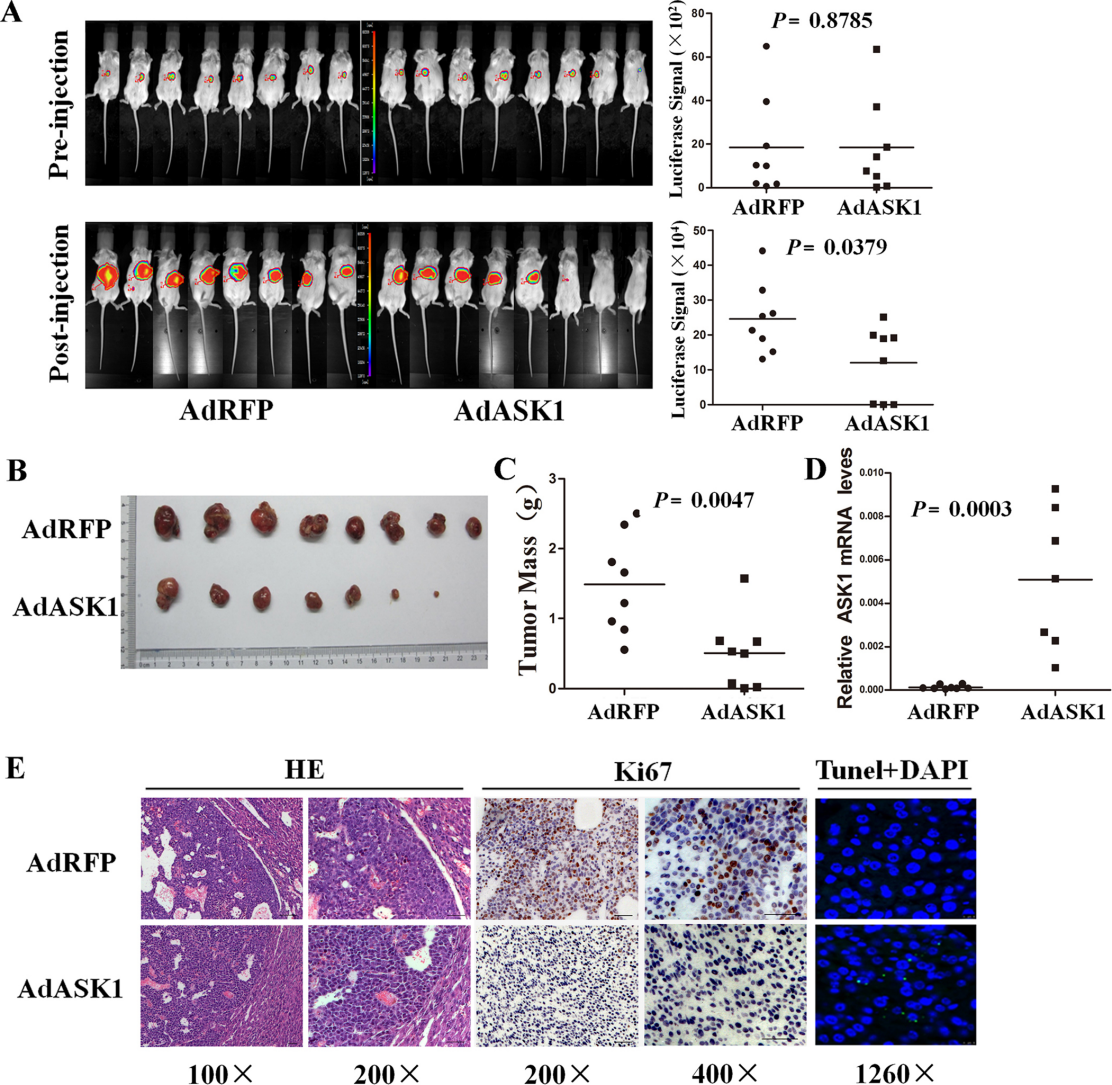
Systemic injection of AdASK1 suppresses orthotopic hepatocellular carcinoma growth (**A**) Images (left) and statistical analysis (right) of luciferase signals of the experiment in BALB/c nude mice transplanted with Huh7 cells stably expressing luciferase. An orthotopic HCC model was established based on implantation of Huh7 tumor piece into the liver, and then AdASK1 or AdRFP injection through tail vein (*n* = 8 in each group). (**B**) Images of tumor nodules with systemically injected AdASK1 or AdRFP at the endpoint. (**C**) Tumor weight measured at the endpoint. (**D**) RT-PCR analysis of ASK1 mRNA expression in tumor nodules systemically injected with AdASK1 or AdRFP. (**E**) Representative images of H & E stained, Ki67 immunohistochemistry and tunel stained of serial sections from tumor nodules systemically injected with AdASK1 or AdRFP. Magnification, ×100, ×200, ×400 and ×1260; Horizontal lines in A, C, and D indicate the median value.

To further determine the antitumor effect on HCC by ASK1, we investigated the inhibitory effect of systemic AdASK1 injection on orthotopic HCC model in NOD/SCID mice. Luciferase-labeled Huh7 cells were injected into nude mice to establish subcutaneous tumors. Subsequently, the tumors were removed and implanted into NOD/SCID mouse liver to establish an orthotopic HCC model. The xenografts had no difference in luciferase expression before adenovirus treatment. AdASK1-injected mice had notably reduced bioluminescence compared with mice that received control virus (Figure 5A). Consistent with this, tumors from AdASK1-treated mice were significantly smaller than those of AdRFP-treated mice (Figure 5B, 5C). ASK1 mRNA levels were significantly increased in AdASK1-treated group compared with the control group (Figure 5D). Consistently, the number of Ki67-positive cells in tumors of ASK1-treated mice was significantly decreased and, conversely, TUNEL-positive cells were increased (Figure 5E and Supplementary Figure S9B). These experiments together reveal that ASK1 has a therapeutic effect on HCC *in vivo*.

### ASK1 inhibits HCC by upregulating p38 phosphorylation

**Figure 6:**
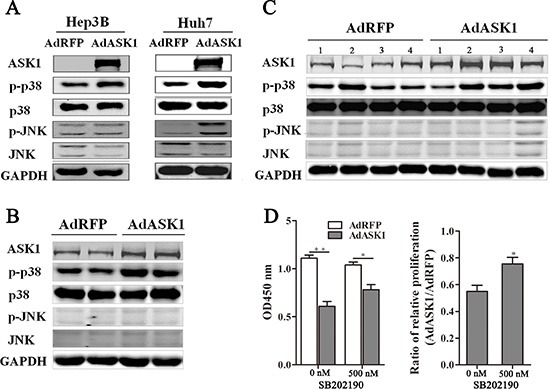
ASK1 overexpression increases p38 phosphorylation (**A**) Western blot analysis of ASK1, p-p38, p38, p-JNK, and JNK expression in AdASK1-infected Hep3B and Huh7 cells. (**B**) Western blot analysis of ASK1, p-p38, p38, p-JNK, and JNK expression in the Huh7 tumor nodules intratumorally injected with AdASK1 or AdRFP. (**C**) Western blot analysis of ASK1, p-p38, p38, p-JNK, and JNK expression in orthotopic HCC model mice systemically injected with AdASK1 or AdRFP. (**D**) Cell proliferation of Hep3B cells incubated with p38 inhibitor SB202190 after AdASK1 infected. Data represent the mean ± SD, *P < 0.05, **P < 0.01.

ASK1 is known to activate both the p38 and JNK pathways, which have been implicated in many cancers. To further investigate the mechanism by which ASK1 suppresses HCC, the expression of ASK1 and several key proteins involved in MAPK signaling were measured by Western blot. As expected, p38 phosphorylation was increased in AdASK1-infected Hep3B and Huh7 cells, but JNK phosphorylation was only increased in AdASK1-infected Huh7 cells (Figure 6A). Consistent with this, Western blot analysis showed that p38 phosphorylation in tumors from both intratumoral injection and systemic delivery with AdASK1 was activated, but no obvious JNK phosphorylation was observed (Figure 6B, 6C). Furthermore, p38 inhibitor SB202190 could partially block ASK1 suppression on HCC cell proliferation (Figure 6D and Supplementary Figure S10). These data reveal that ASK1 may suppress the malignant properties of HCC via increasing p38 phosphorylation.

### ASK1 mediates the suppression of HCC by HNF4α

**Figure 7:**
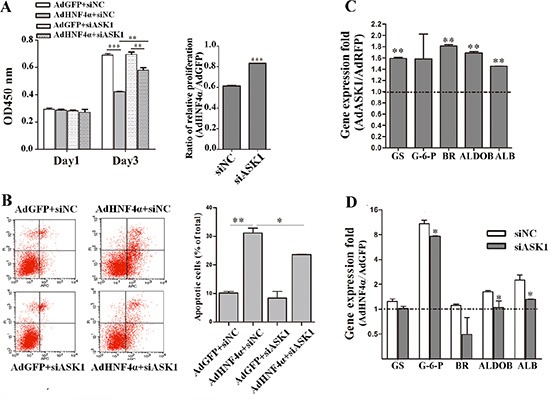
HNF4α suppresses HCC tumorigenicity partly through ASK1 upregulation (**A**) Hep3B cells infected with AdHNF4α or AdGFP were transfected with siASK1 or siNC. Cell proliferation was measured on day 1 and 3 after transfection. (**B**) Flow cytometric analysis of Hep3B cells infected with AdHNF4α or AdGFP and then transfected with siASK1 or siNC. (**C**) Gene expression folds of some hepatocyte marker genes in AdASK1-infected Hep3B cells versus the AdRFP-infected group. Significant increases in expression of characteristic hepatocyte markers included glutamine synthetase (GS), biliverdin reductase (BR), aldolase B (ALDOB) and albumin (ALB). (**D**) RT-PCR analysis of mRNA expression for some hepatocyte marker genes in HNF4α-overexpressing and siASK1-transfected Hep3B cells. Significant difference were observed in expression of glucose-6-phosphatase (G-6-P), ALDOB and ALB. Each value represents the relative ratio of AdHNF4α to AdGFP. Data represent the mean ± SD, *P < 0.05, **P < 0.01, ***P < 0.001.

Given that HNF4α overexpression activated ASK1 and that ASK1 suppressed the malignant phenotypes of HCC cells, we next determined whether ASK1 mediated the effect of HNF4α on HCC by a combined loss-of-function and gain-of-function approach. HCC cells overexpressing HNF4α were post-transfected with siASK1 or siNC (Supplementary Figure S11A). As expected, silencing ASK1 expression blocked HNF4α inhibition of HCC cell proliferation (Figure 7A). Consistently, repression of cell proliferation by ectopic HNF4α could be partly reversed by SB202190 (Supplementary Figure S11B). Similarly, flow cytometry showed that the promoting of cell apoptosis by HNF4α was partially reversed after ASK1 depletion (Figure 7B and Supplementary Figure S11C). A previous study from our laboratory reported that HNF4α re-established the expression profile of characteristic hepatocyte markers in hepatoma cells [13]. Interestingly, ectopic ASK1 expression also promoted the re-expression of some of these hepatocyte marker genes (Figure 7C), seemed to mimic the function of HNF4α. Conversely, the re-expression of hepatocyte marker genes by ectopic HNF4α was also blocked by siASK1 in Hep3B cells (Figure 7D). Taken together, these data indicate that the HNF4α inhibition of HCC can be, at least partially, attributed to ASK1 upregulation.

## DISCUSSION

A previous study found that ASK1 expression was lower in breast cancer tissues than in normal tissues [35], suggesting that ASK1 has a tumor-suppressing function. In the current study, we proved that low ASK1 expression is correlated with poor prognosis for HCC patients. More interestingly, the malignant properties of HCC cells were also dramatically suppressed by ASK1 overexpression. Furthermore, ASK1 administration showed a dramatic antitumor effect *in vivo*. These data imply the clinical significance of ASK1 in HCC patients and present a novel target for HCC therapy.

HNF4α is a key transcriptional regulator involved in HCC [9, 10]. Although some mechanisms involved in the therapeutic effect of HNF4α on HCC have been comprehended in depth over the past few years [9, 12, 15, 16], it is still of considerable interest to identify new pathways, as HNF4α is a wide-acting transcription factor that regulates numerous downstream targets [36, 37]. In this study, a cluster of genes in pathways linked to distinct categories were affected by HNF4α overexpression in HCC cells. Specifically, we demonstrated that HNF4α could transcriptionally upregulate *ASK1* by directly targeting its promoter in HCC cells. More importantly, ASK1 downregulation partially abrogated HNF4α inhibition of HCCs. These data suggest that ASK1 contributes to HNF4α-mediated HCC differentiation, which further elucidates and enriches the mechanism of HNF4α on HCC.

ASK1 has been linked to a variety of cellular functions and pathophysiological processes, such as proliferation, survival, and the inflammatory response [38, 39]. Most importantly, the role of ASK1 in cell differentiation has been studied with great interest in recent years [21, 22, 40–42]. Previous studies have shown that ASK1 was involved in the differentiation process not only of stem/progenitor cells but also of terminally differentiated cells [19, 21, 41], suggesting the core role of ASK1 on differentiation. However, these studies merely found altered ASK1 expression during the cell differentiation process. There is no direct evidence to certify the participation of ASK1 in cell differentiation, especially in HCC cell differentiation. Herein we report that ASK1 inhibited the malignant properties of HCC cells. More intriguingly, and consistent with the effect of HNF4α, ectopic ASK1 expression also re-established the normal expression profile of hepatocyte marker genes. Together, these findings first demonstrate the effect of ASK1 on HCC differentiation, which extends our knowledge of ASK1 in cancer. Whether ASK1 has similar differentiation action on other tumors is worth further evaluation.

The p38 MAPKs is predominantly activated through its phosphorylation by ASK1. Activated p38 was originally considered part of a classic proapoptotic pathway [26, 43, 44]. Nevertheless, substantial evidence has also established a unique role for p38 MAPK in cell differentiation [41, 42, 45–48]. With this study, p38 pathway was obviously activated by ASK1. Moreover, the p38 inhibitor partially antagonized the effect of ASK1 or HNF4α overexpression on cell proliferation. Thus, we could conclude that the p38 pathway participate the HNF4α-induced HCC differentiation. In addition to p38, the JNK pathway, another downstream pathway of ASK1, also has an important role in mesenchymal stem cell differentiation [49]. Whether JNK was participated in this process need to be further studied in future.

In conclusion, the present work first reports the prognostic value of ASK1 in HCC patients and certifies its differentiation and suppressive effect on HCC. These data further broaden our understanding of ASK1 biological function and suggests a novel target for HCC therapy.

## MATERIALS AND METHODS

More details are presented in the Supplementary Materials and Methods.

### Adenoviral vectors

The recombinant adenoviruses AdHNF4α and the control virus AdGFP were previously constructed in our laboratory [13]. The ASK1 expression plasmid was obtained from the DNA Resource Core of Harvard University. Replication-deficient recombinant adenoviruses AdASK1 and the AdRFP control were constructed by Shanghai Sunbio Medical Biotechnology (Shanghai, China).

### cDNA microarray analysis

Hep3B cells were harvested 72 h after infection with AdHNF4α or AdGFP at a multiplicity of infection of 100. Total RNA was extracted and used to prepare cDNA probes. cDNA microarrays (Affymetrix U133 plus 2.0 Array) were used to generate differential gene expression profiles, and image processing and data analysis were performed using Affymetrix GeneChip Scanner 3000 and GeneChip Operating Software. The differential genes were subjected to the KEGG pathway analysis performed with DAVID software [33].

### Patient tissue specimens and microarray analysis

A total of 60 HCC tissue samples paired with surrounding noncancerous tissues were obtained from patients who underwent surgical resection at the Eastern Hepatobiliary Surgery Hospital and Changzheng Hospital (Shanghai, China). HNF4α and ASK1 expression were measured by RT-PCR. A tissue microarray block containing 90 HCCs along with case-matched noncancerous surrounding tissue samples was constructed using a tissue microarrayer. Immunostaining of tissue microarray slides was performed using an anti-ASK1 antibody (CST, Boston, MA, USA). Nuclear ASK1 staining was assessed on a four-point scale (negative, 1; weakly positive, 2; positive, 3; strong positive, 4) according to the percentage of positively stained cells and the nuclear staining intensity. The immunostaining score was obtained by multiplying the nuclear staining intensity by the percentage of positively stained cells. OS refers to the time to death after surgery. Written informed consent and approval by the Ethics Committee of Second Military Medical University, Shanghai, China, were obtained for all human experiments.

### Chromatin immunoprecipitation assays

ChIP assays were performed essentially as previously described [15]. Briefly, Hep3B cells were cross-linked and processed according to the ChIP Assay Kit protocol (Millipore, Boston, USA). Anti-HNF4α primary antibody or control IgG was used for immunoprecipitation experiments. RT-PCR analysis was carried out for HNF4α binding sites in *ASK1* promoter. The predicted HNF4α-binding sites on *ASK1* gene were analyzed by JASPAR, a high-quality transcription factor binding profile database [34]. The sequence of the putative binding site of HNF4α in *ASK1* promoter and RT-PCR primers are listed in Supplementary Figure S3 and Table S1. At least three independent experiments were performed.

### Luciferase reporter assay

To assess the effect of HNF4α expression on *ASK1* promoter activity, the *ASK1* promoter sequence containing the predicted HNF4α-binding sites was PCR amplified from Hep3B genomic DNA and cloned into the pGL3-promoter luciferase vector. A mutated reporter carrying disrupted binding sites was generated by PCR-directed mutagenesis.

Hep3B and Huh7 cells growing in 24-well plates were first infected with HNF4α adenovirus and then transfected with *ASK1* promoter vectors together with the control pRL-SV40 vector. Luciferase activity was measured using the Dual-Glo Luciferase Assay System (E2920; Promega) at 48 h post-transfection. At least three independent experiments were carried out for each condition.

### Cell proliferation and colony formation assays

To test the inhibitory effect of ASK1 on HCC cell proliferation, transfected or infected HCC cells were plated into 24-well plates overnight and then seeded at 4 × 10^3^/well into triplicate wells of a 96-well plate. Cell proliferation was assessed using Cell Counting Kit-8 (Dojindo, Tokyo, Japan). For colony formation assays, Hep3B and Huh7 cells infected or transfected for 6 h were seeded into 60-mm dishes and grown in culture medium for approximately 2 weeks. Colonies were then fixed with 4% paraformaldehyde and visualized by crystal violet staining.

### Flow cytometry

To quantify cellular apoptosis, adenovirus-infected HCC cells were stained with an annexin V staining Kit (Bestbio, Beijing, China) according to the manufacturer's protocol and analyzed by flow cytometry. Apoptotic cells were defined as annexin-V-positive cells. Three independent experiments were carried out.

### Migration and invasion assay

For cell migration and invasion assays, transfected or infected cells were seeded at 5 × 10^4^/transwell into the upper chamber (BD Bioscience, New Jersey, USA) with or without matrigel-coated membrane under serum-free conditions. The bottom chamber was contained by medium supplemented with 10% fetal bovine serum. After incubation for 24 or 48 h, cells in the upper chamber were carefully removed and cells adhering to the underside of the membrane were stained with crystal violet and at least five fields per sample were photographed under inverted microscopy. Cell migration and invasion were measured using image analysis software (Image-Pro Plus 6.0; Media Cybernetics). At least three independent experiments were performed for each condition.

### Animal models

Male BALC/c nude mice or NOD/SCID mice of 4–6 weeks of age were used in this study (Shanghai SLAC Laboratory Animal Company). To detect the effect of ASK1 overexpression on HCC cell tumorigenicity, Hep3B or Huh7 cells infected with AdASK1 or AdRFP were inoculated subcutaneously into both flanks of each BALB/c nude mouse (*n* = 6). Tumor formation, size (volume = 0.5 × width^2^ × length), and final weight were assessed. To investigate the suppressive effect of ASK1, a subcutaneously implanted HCC model was established by injecting 2 × 10^6^ Huh7 cells into the armpits of nude mice. When the tumor volume reached about 200 mm^3^, mice were randomly assigned to treatment and control groups, and intratumorally injected with AdASK1 or AdRFP twice a week for up to 3 weeks. The kinetics of tumor formation was estimated. At the time of euthanasia, tumors were removed for further analysis.

To further explore the effect of ASK1 overexpression *in vivo*, Huh7 cells labelled with luciferase gene were used to establish subcutaneous tumors, as previously described [16, 50]. Once each tumor reached 500 mm^3^, it was removed and cut into 1-mm^3^ pieces. Subsequently, tumor pieces were implanted into the livers of NOD/SCID mice to mimic primary HCCs. After a week, mice were assigned into two groups matched according to luciferase burden; hereafter, AdASK1 or AdRFP was injected into the tail vein twice a week for up to 3 weeks. Bioluminescence was measured after intraperitoneal injection with D-luciferin (5 μL/g body weight) and bioluminescent images were acquired using the NightOWL imaging system (Berthold LB983, Berthold Technologies, Germany).

All animal experiments were performed at the Second Military Medical University according to protocols approved by the institutional animal care committee.

### Immunohistochemistry and TUNEL assay

Tissue samples were fixed in formaldehyde solution and embedded in paraffin, and then serial sections were cut. Tissue samples were subjected to hematoxylin and eosin (H & E) staining for standard histologic examination and to immunohistochemical examination for HNF4α, ASK1, p-ASK1 and Ki67 expression. For TUNEL staining, paraffin-embedded tissue samples were analyzed using a One Step TUNEL Apoptosis Assay Kit (Beyotime, Jiangsu, China). Images were acquired using a Leica confocal microscope and analyzed with dedicated software (Leica Microsystems).

### Statistical analysis

Statistical analysis was performed using SPSS software (16.0 version) with a *P* < 0.05 considered significant. Student's *t*-tests were used to analyze experimental data involving only two groups. Statistical comparisons of more than two groups were evaluated using one-way analysis of variance. Unequal variances pairing were performed using the Wilcoxon signed rank test or the Mann-Whitney *U* test. The chi-square test was used to compare two sample rates. The Kaplan-Meier method was used to calculate the survival time, and its significance was determined with log-rank test. *P < 0.05; **P < 0.01; ***P < 0.001.

## SUPPLEMENTARY MATERIALS FIGURES AND TABLES


