# Supplementary material for: Apoptosis signal-regulating kinase 1 mediates the inhibitory effect of hepatocyte nuclear factor-4α on hepatocellular carcinoma
Source: Oncotarget. 2016 Mar 30;7(19):27408–21. doi: 10.18632/oncotarget.8478 (PMC5053659; doi:10.18632/oncotarget.8478)
Supplement: Supplementary file 1 [file oncotarget-07-27408-s001.pdf]

Apoptosis signal-regulating kinase 1 mediates the inhibitory effect of hepatocyte nuclear factor-4α on hepatocellular carcinoma

Supplementary Materials

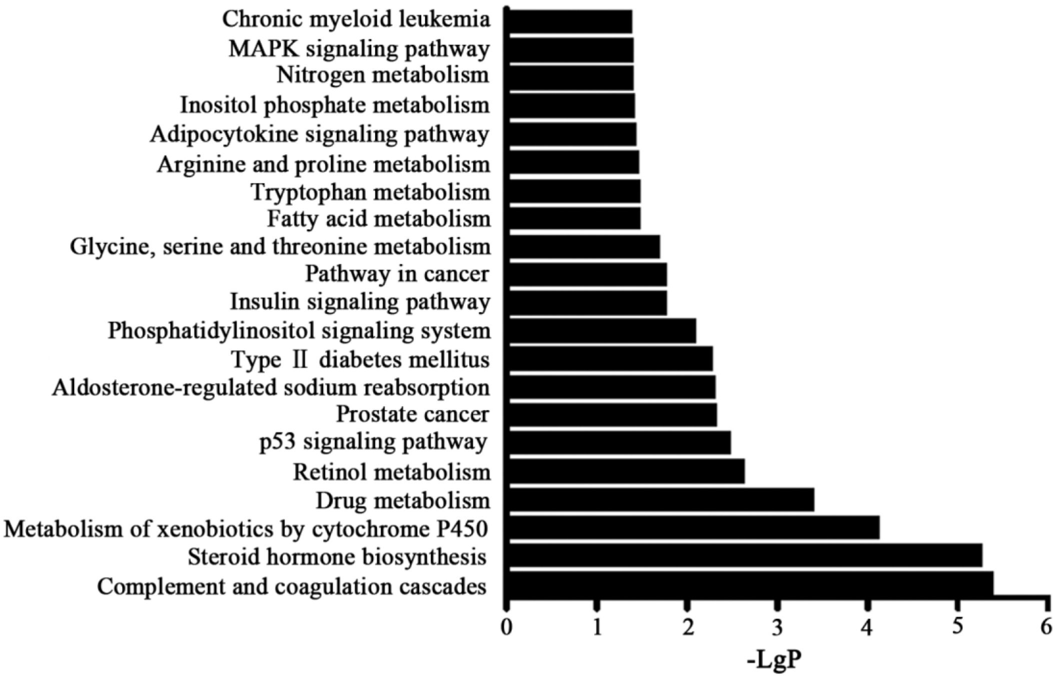

Supplementary Figure S1: HNF4α regulates MAPK signaling pathways. Pathway analysis was based on the differential gene expression obtained by cDNA microarray analysis of Hep3B cells infected with AdHNF4α or AdGFP for 72 h. Statistical significance was set at a P value < 0.05 for pathway categories. The vertical axis is the pathway category and the horizontal axis is the minus common logarithm of the P value (−LgP).

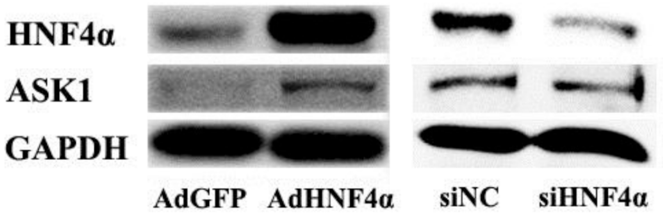

Supplementary Figure S2: HNF4α regulates ASK1 expression. Western blot analysis of ASK1 expression in AdHNF4α-infected Huh7 cells.

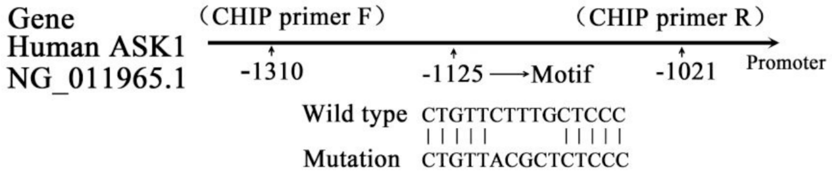

Supplementary Figure S3: HNF4α regulates ASK1 by binding to its promoter. Schematic representation of the predicted target HNF4α-binding regions in the *ASK1* promoter and the corresponding mutated sequences.

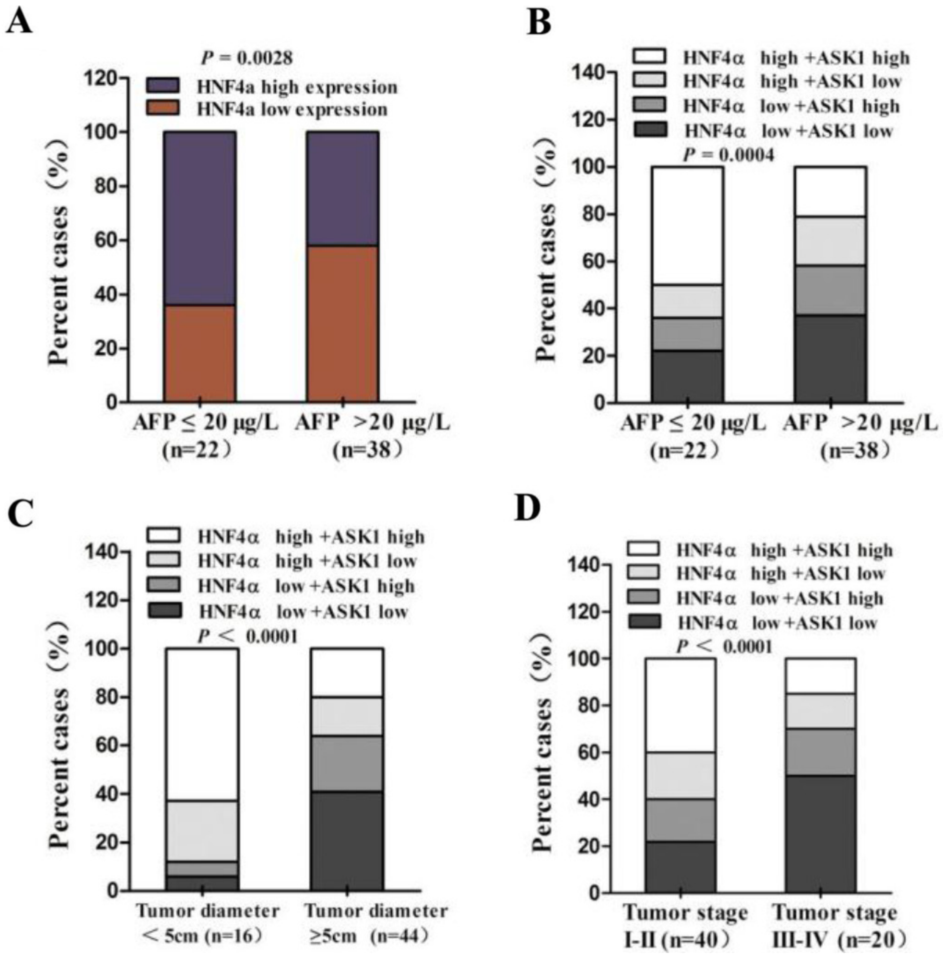

Supplementary Figure S4: HNF4α and ASK1 expression with HCC phenotype in clinical samples. (**A**) Reduced HNF4α mRNA expression was more common in HCC samples with high AFP mRNA levels than in those with low AFP mRNA levels. Low level: AFP ≤ 20 μg/L, n = 22; high level: AFP > 20 μg/L, n = 38. (**B–D**) Reduced HNF4α and ASK1 mRNA expression were more common in samples from HCC with more aggressive pathological features, including high AFP(B), larger tumor size (C) and advanced tumor stage (D).

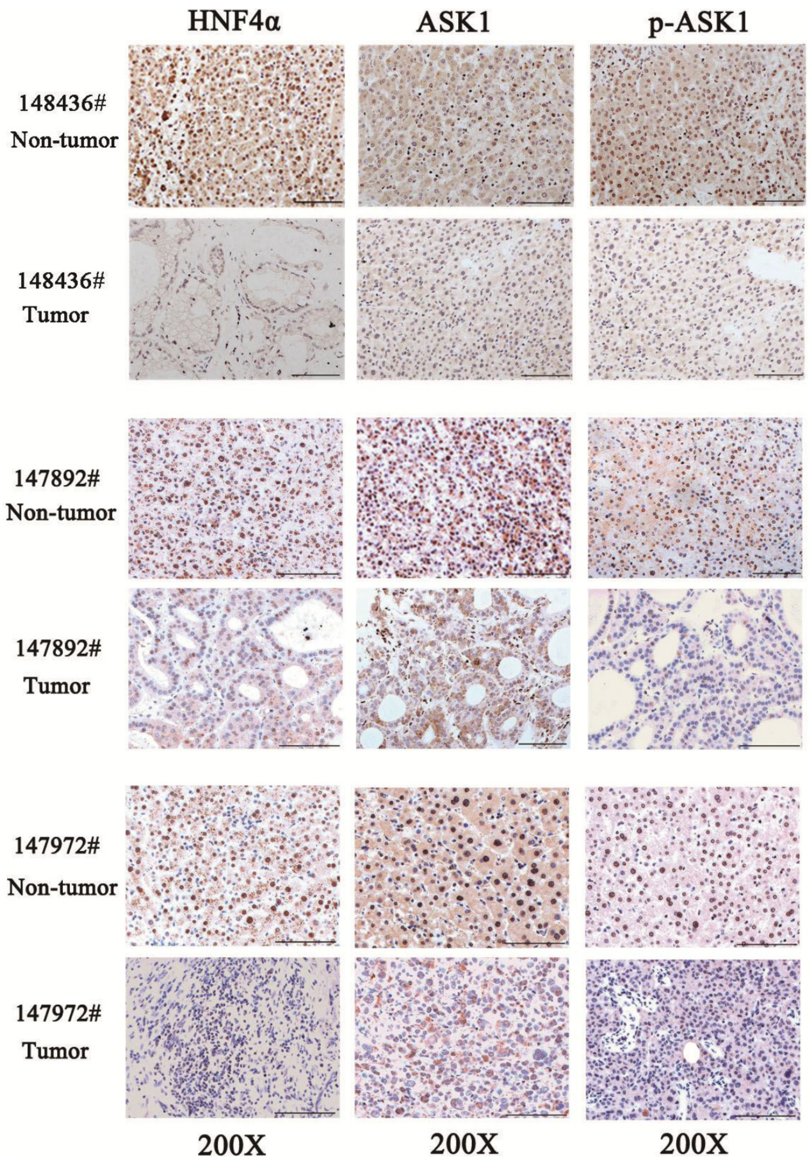

Supplementary Figure S5: HNF4α and ASK1 expression in clinical samples. Images of HNF4α, ASK1, p-ASK1 immunohistochemistry of serial sections from three HCC patients with non-tumor tissue or tumor tissue. Magnification, ×200.

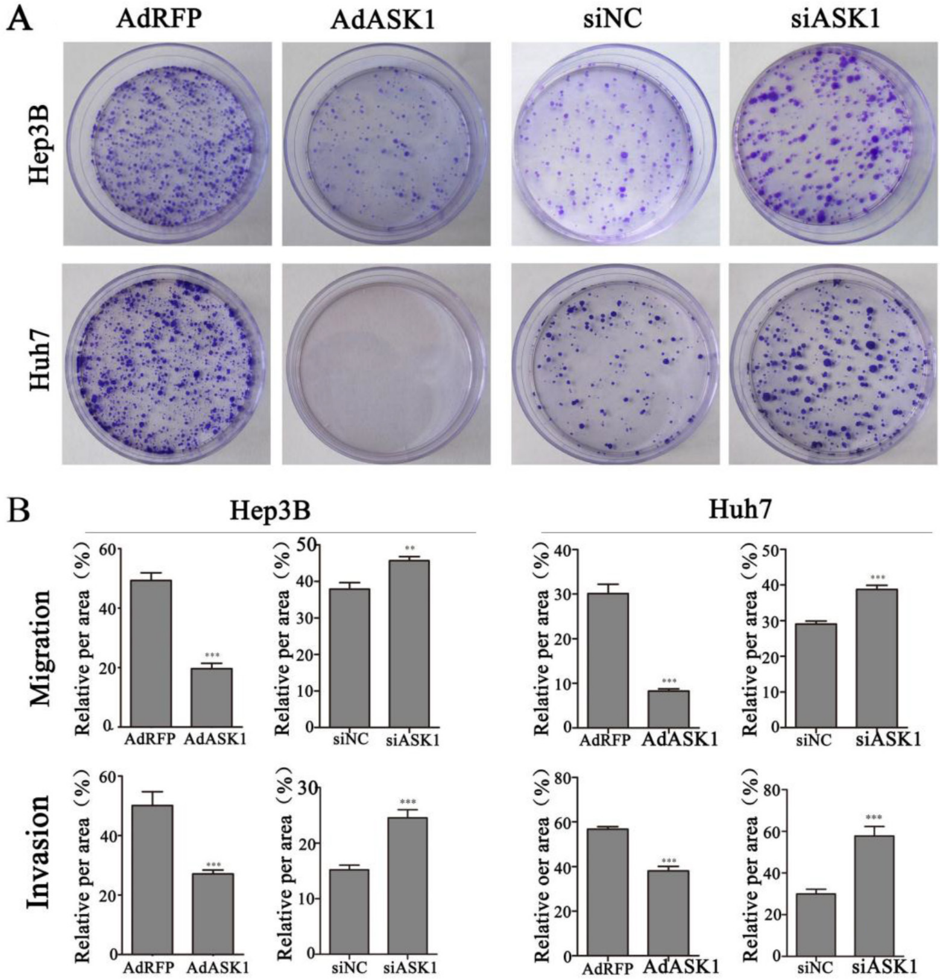

Supplementary Figure S6: ASK1 inhibits the malignant properties of HCC cells *in vitro*. (**A**) Images of colony formation assays performed using Hep3B and Huh7 cells infected with AdASK1 or AdRFP or transfected with siNC or siASK1. (**B**) Migration and invasion of Hep3B and Huh7 cells infected with AdASK1 or AdRFP or transfected with siNC or siASK1.The percentage of cells passing through the transwell membranes was calculated and compared in the corresponding diagrams shown under the images. ***P* < 0.01, ****P* < 0.001.

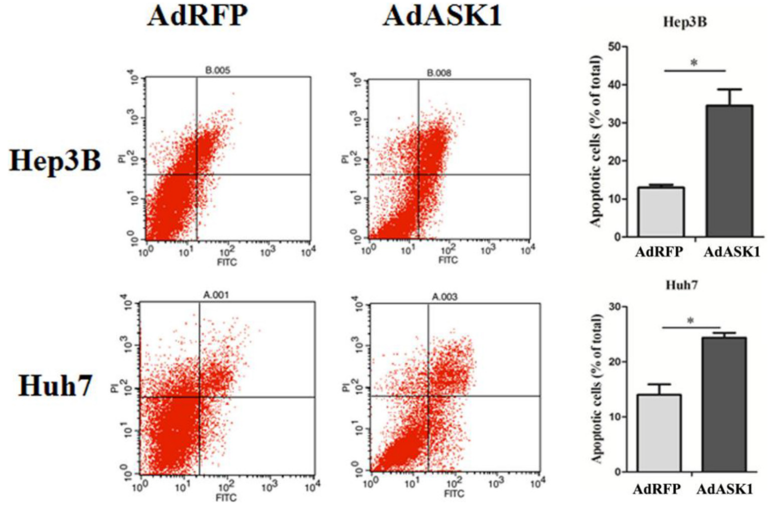

Supplementary Figure S7: ASK1 overexpression increases cell apoptosis *in vitro*. Flow cytometric analysis was used to evaluate apoptosis in Hep3B and Huh7cells infected with AdASK1 or AdRFP. **P* < 0.05.

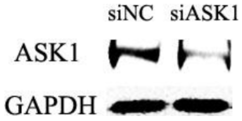

Supplementary Figure S8: ASK1 expression in siASK1-transfected Hep3B cells. Western blot analysis of ASK1 expression in siASK1-transfected Hep3B cells.

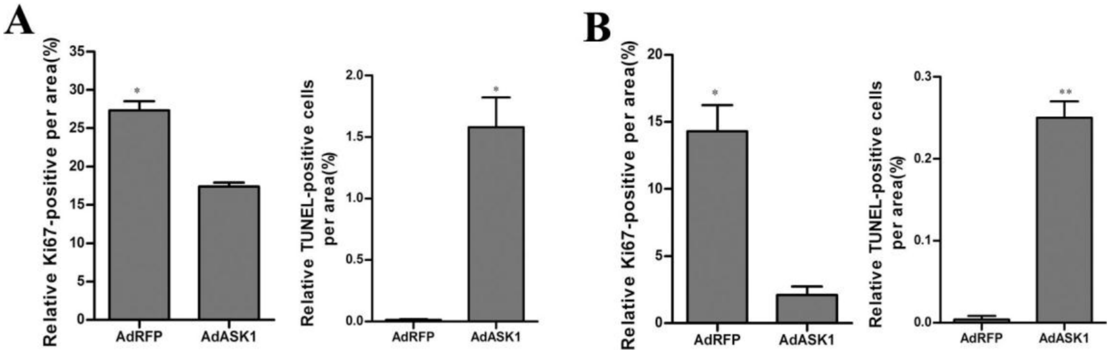

Supplementary Figure S9: Statistical analysis of Ki67 and TUNEL positive in mice. (**A**) The quantified Ki67 positive cells and TUNEL positive cells in intratumoral injection model. (**B**) The quantified Ki67 positive cells and TUNEL positive cells in orthotopic HCC model. *$P < 0.05$,**$P < 0.01$.

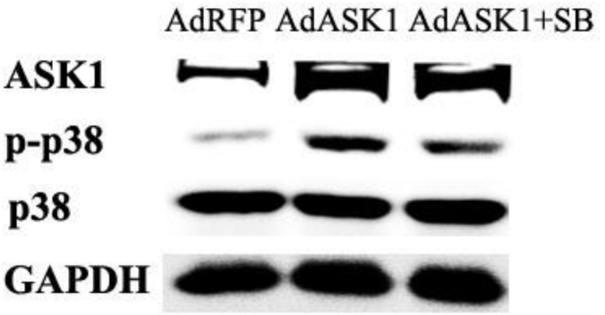

Supplementary Figure S10: The expression of p38 analyzed by Western blot. Western blot analysis of p-p38 and p38 expression in Hep3B cells incubated with p38 inhibitor SB202190 after AdASK1 infected.

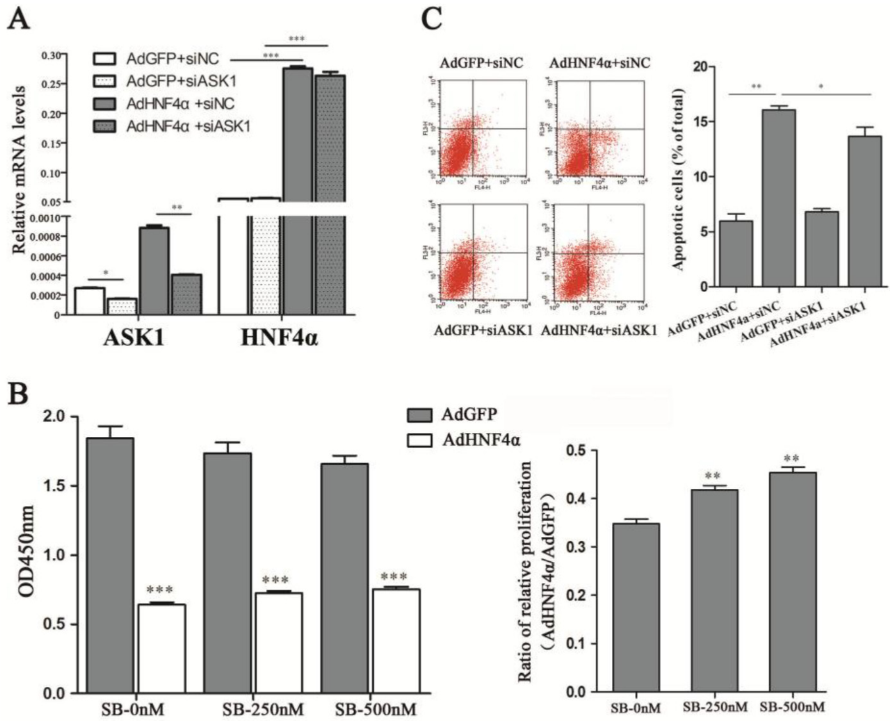

Supplementary Figure S11: ASK1 overexpression reverses the suppressive effects of HNF4α on HCC. (**A**) ASK1 and HNF4α mRNA levels in Hep3B cells infected with AdHNF4α or AdGFP and then transfected with siNC or siASK1. **P* < 0.05, ***P* < 0.01, ****P* < 0.001. (**B**) YY-8103 cells were infected with AdHNF4α or AdGFP for 48 h and then treated with the p38 MAPK inhibitor SB 202190 for 24 h. Cell proliferation was then measured. Each value represents the mean ± SD of triplicate experiments. Left:, original data; right, the relative ratio in AdHNF4α-infected versus AdGFP-infected cells. (**C**) Flow cytometric analysis of Huh7 cells infected with AdHNF4α or AdGFP and then transfected with siASK1 or siNC. **P* < 0.05, ***P* < 0.01.

Supplementary Table S1: Oligonucleotides used in real-time PCR, cloning and knockdown studies

gene	Forward primer (5′- 3′)	Reverse primer (5′- 3′)	Product size (bp)
**Primers for Real time PCR**			
**ASK1**	TGAGGAACAGCCTTCAAATCAA	TCACTCTCAGCCAGTCGGTAAG	365
**HNF4α**	CTTCCTTCTTCATGCCAG	ACACGTCCCCATCTGAAG	271
**GS**	CCTGCTTGTATGCTGGAGTC	GAAAAGTCGTTGATGTTGGA	396
**G-6-P**	GGCTCCATGACTGTGGGATC	TTCAGCTGCACAGCCCAGAA	475
**BR**	ACAAGGTGCTGCGGGAATCA	ACTGGTGGGAGGGGTAGGTG	209
**ALDOB**	AGGAGGACTCTTCTCTCCCAA	GATTCATCTGCAGCCAGGAT	141
**ALB**	AGCCTAAGGCAGCTTGACTT	CTCGATGAACTTCGGGATGA	1210
**β-actin**	CCTGGCACCCAGCACAAT	GGGCCGGACTCGTCATACT	144
**siRNA and miRNA sequences**			
**si-NC**	UUCUCCGAACGUGUCACGUTT	ACGUGACACGUUCGGAGAATT	
**si-HNF4α**	CCACAUGUACUCCUGCAGATT	UCUGCAGGAGUACAUGUGGTT	
**si-ASK1**	GGUAUACAUGAGUGGAAUUTT	AAUUCCACUCAUGUAUACCTT	
**Primers for plasmid construction**			
**ASK1-promoter**	CGACGCGTGCATATCACCTG AGGTCAGGA (Mlu I)	CTAGCTAGCGATAGAAGATGG AGTCTCTACCCG (Nhe I)	534
**ASK1-promoter-mutation**	TGGGAACTTACACATCTGTTAC GCTCTCCCCAAATAAGCAGGTA	TACCTGCTTATTTGGGGAGAGC GTAACAGATGTGTAAGTTCCCA	
**Primers for CHIP**			
**HNF4α on ASK1 promoter**	AGAGCGAAACTCCGTCTCAAAA	CCAGTGCAATAAGGGGACAAAG	311

Supplementary Table S2: Clinicopathologic correlation of ASK1 downregulation in human HCCs

Variables	ASK1			*P* Value (χ^2 test)
	**All cases (*n* = 60)**	**High expression (*n* = 30)**	**Low expression (*n* = 30)**	
Age (years)*				
< 55	36	17	19	0.301
≥ 55	24	13	11	
Sex				
Male	52	27	25	0.396
Female	8	3	5	
HBsAg				
Negative	11	6	5	0.697
Positive	49	24	25	
Liver cirrhosis				
Yes	22	8	14	0.068
No	38	22	16	
Tumor size (cm)				
≤ 5	16	11	5	0.034
> 5	44	19	25	
Tumor stage (TNM)				
I	22	11	11	0.009
II	18	12	6	
III	19	7	12	
IV	1	0	1	
Tumor encapsulation				
Present	24	16	8	0.013
Absent	36	14	22	
Tumor microsatellite				
Absent	34	19	15	0.231
Present	26	11	15	
Venous invasion				
Absent	30	15	15	1.0
Present	30	15	15	

*Mean age.

Supplementary Table S3: Clinicopathologic correlation of HNF4α downregulation in human HCCs

Variables	HNF4α			*P* Value (χ^2 test)
	All cases (*n* = 60)	High expression (*n* = 30)	Low expression (*n* = 30)	
Age (years)*				
< 55	36	10	14	0.227
≥ 55	24	20	16	
Sex				
Male	52	25	27	0.363
Female	8	5	3	
HBsAg				
Negative	11	6	5	0.697
Positive	49	24	25	
Liver cirrhosis				
Yes	22	10	12	0.539
No	38	20	18	
Tumor size (cm)				
≤ 5	16	14	2	0.0001
> 5	44	16	28	
Tumor stage (TNM)				
I	22	11	11	0.022
II	18	13	5	
III	19	6	13	
IV	1	0	1	
Tumor encapsulation				
Present	24	12	12	1.0
Absent	36	18	18	
Tumor microsatellite				
Absent	34	17	17	1.0
Present	26	13	13	
Venous invasion				
Absent	30	15	15	1.0
Present	30	15	15	

* Mean age.

MATERIALS AND METHODS

Cell culture and small interfering RNAs

The human HCC cell lines Hep3B and Huh7 were cultured in Minimum Essential Medium (MEM; Gibco, Grand Island, NY, USA) and Dulbecco's modified Eagle medium (DMEM; Gibco) plus heat-inactivated fetal bovine serum (FBS) in a 5% CO_2 atmosphere. Small interfering RNA (siRNA) targeting human *ASK1* (Stealth RNAi siRNA duplex) or negative-control siRNA duplexes were purchased from GenePharma (Shanghai, China). All siRNA sequences are listed in Supplementary Table S1.

Real-time polymerase chain reaction (RT-PCR)

Total RNA was extracted from cells and tissues using the Trizol reagent (Takara, Kyoto, Japan). RNA was reverse transcribed to form cDNA using oligo (dT) primers and M-MLV reverse transcriptase according to the manufacturer's instructions (Takara). Specific transcripts were quantified in triplicate by real-time RT-PCR with a SYBR Green PCR Kit (Takara). The relative expression of specific genes was normalized to the housekeeping gene β-actin. Quantitative RT-PCR primers are listed in Supplementary Table S1.

Western blot analysis

Proteins were extracted using RIPA buffer (P0013B, Beyotime, Suzhou, China) supplemented with protease inhibitor cocktail (Merck), separated by sodium dodecyl sulfatepolyacrylamidegel electrophoresis (SDS-PAGE), and transferred to nitrocellulose membrane (HAHY00010, Millipore). Membranes were blocked in PBST containing 5% non-fat milk, and then probed with specific primary antibodies, followed by incubation donkey-anti-goat or donkey-anti-rabbit secondary antibody (IRDye 700 or IRDye 800, respectively). Protein bands were detected with Odyssey Infrared Imaging System (LI-COR, Nebraska, USA). Equal loading was confirmed by GAPDH immunoblotting. Primary antibodies for western blotting were rabbit anti-human phospho-ASK1(1:500, CST, Boston, MA, USA), rabbit anti-human ASK1 (1:500, CST), rabbit anti-human phospho-p38 MAPK (1:1000, CST), anti-p38 MAPK (1:1000, CST), rabbit anti-human phospho-JNK (1:1000, CST), rabbit anti-human JNK (1:1000, CST), goat anti-human HNF4α (1:500, Santa Cruz), and GAPDH (1:5000, KangChen, Guangzhou, China).
